# Improved normalization of species count data in ecology by scaling with ranked subsampling (SRS): application to microbial communities

**DOI:** 10.7717/peerj.9593

**Published:** 2020-08-03

**Authors:** Lukas Beule, Petr Karlovsky

**Affiliations:** Molecular Phytopathology and Mycotoxin Research, Georg-August Universität Göttingen, Göttingen, Germany

**Keywords:** Species count, Microbiome, Amplicon sequencing, Count data normalization, Scaling with ranked subsampling (SRS), Rarefying, Alpha diversity, Beta diversity, Relative abundance

## Abstract

**Background:**

Analysis of species count data in ecology often requires normalization to an identical sample size. Rarefying (random subsampling without replacement), which is the current standard method for normalization, has been widely criticized for its poor reproducibility and potential distortion of the community structure. In the context of microbiome count data, researchers explicitly advised against the use of rarefying. Here we introduce a normalization method for species count data called scaling with ranked subsampling (SRS) and demonstrate its suitability for the analysis of microbial communities.

**Methods:**

SRS consists of two steps. In the scaling step, the counts for all species or operational taxonomic units (OTUs) are divided by a scaling factor chosen in such a way that the sum of scaled counts equals the selected total number of counts C_min_. The relative frequencies of all OTUs remain unchanged. In the subsequent ranked subsampling step, non-integer count values are converted into integers by an algorithm that minimizes subsampling error with regard to the population structure (relative frequencies of species or OTUs) while keeping the total number of counts equal C_min_. SRS and rarefying were compared by normalizing a test library representing a soil bacterial community. Common parameters of biodiversity and population structure (Shannon index *H’*, species richness, species composition, and relative abundances of OTUs) were determined for libraries normalized to different size by rarefying as well as SRS with 10,000 replications each. An implementation of SRS in R is available for download (https://doi.org/10.20387/BONARES-2657-1NP3).

**Results:**

SRS showed greater reproducibility and preserved OTU frequencies and alpha diversity better than rarefying. The variance in Shannon diversity increased with the reduction of the library size after rarefying but remained zero for SRS. Relative abundances of OTUs strongly varied among libraries generated by rarefying, whereas libraries normalized by SRS showed only negligible variation. Bray–Curtis index of dissimilarity among replicates of the same library normalized by rarefying revealed a large variation in species composition, which reached complete dissimilarity (not a single OTU shared) among some libraries rarefied to a small size. The dissimilarity among replicated libraries normalized by SRS remained negligibly low at each library size. The variance in dissimilarity increased with the decreasing library size after rarefying, whereas it remained either zero or negligibly low after SRS.

**Conclusions:**

Normalization of OTU or species counts by scaling with ranked subsampling preserves the original community structure by minimizing subsampling errors. We therefore propose SRS for the normalization of biological count data.

## Introduction

Species counts are fundamental data in studies of ecology and biological diversity. A specific kind of count data, used in studies of the microbiome using next generation sequencing (NGS), are counts of nucleotide sequences that represent operational taxonomic units (OTUs). The so-called amplicon sequencing by NGS became the key technique for the exploration of microbial communities inhabiting diverse environments, such as deep-sea sediments (e.g., Sogin et al., 2006), soils (e.g., Gilbert, Jansson & Knight, 2014), and the human gut (e.g., Yatsunenko et al., 2012). Amplicon sequencing by NGS is also increasingly popular in studies of invertebrate diversity (Hajibabaei et al., 2011; Carew et al., 2013; Morinière et al., 2016; Vivien, Lejzerowicz & Pawlowski, 2016). Accumulation of these data motivated the development of bioinformatic tools and pipelines for their processing. Recently, it has been shown that the choice of bioinformatic tools can affect the results and, in some cases, even lead to different interpretation of the results (Siegwald et al., 2019). Therefore, the choice of analysis tools should be taken into account when comparing microbiome studies (Allali et al., 2017).

In studies of microbial communities by NGS, samples are represented by libraries, which consist of DNA fragments amplified by PCR and attached to adapters required for the sequencing. Multiplex sequencing, which is sequencing pooled libraries, in a single sequencing run, is widely used to lower sequencing costs. A disadvantage of multiplexing is that the number of sequences obtained per library (sample) can span orders of magnitude (McMurdie & Holmes, 2014). Comparative analysis requires identical sample size, therefore microbiome count data are commonly normalized to the same total count per library. Over half a century ago, Sanders (1968) proposed random subsampling without replacement, designated ‘rarefying’, to this end. Since then, rarefying has been used for the normalization of species count data in ecology as well as for NGS data in microbiology. For libraries with counts above a selected threshold, a subsample from each library is generated by randomly picking reads without replacement until the selected number of counts is reached. Although rarefying has become the standard tool in microbiome data analysis (Weiss et al., 2017), its disadvantages have been recognized (McMurdie & Holmes, 2014; Weiss et al., 2017; Willis, 2019). For example, McMurdie & Holmes (2014) demonstrated that rarefying is statistically inadmissible and should not be used. More recently, Willis (2019) pointed at the strong bias in alpha diversity estimates for unequal or rarefied microbiome count data. This is because rare OTUs may be overrepresented or underrepresented in libraries normalized to a small size by rarefying. The growing number of normalization methods indicates that the issue of normalization has not been conclusively solved yet. It is evident that any kind of normalization leads to loss of information that should be avoided if possible. Most diversity indices and statistical tests used in community analysis however do not account for the effect of library size; therefore they require normalization of libraries to the same size.

An alternative normalization to rarefying is scaling, which adjusts the size of all samples to the same value by multiplying the counts by a constant factor. Simple scaling preserves the relative frequencies of OTUs but it also keeps the number of OTUs unchanged, preserving the disparity between large and small libraries: a larger number of sampled individuals or sequence reads likely contains a larger number of species or OTUs and thus possess higher alpha diversity (e.g., species richness). Simple scaling does not compensate for the effect of the sample size or library size on species richness. In the analysis of microbial communities, differences in library size mainly originate from unequal pooling of PCR products prior to sequencing. In order to pool PCR products from individual samples in equimolar amounts (e.g., Kozich et al., 2013), DNA concentrations are commonly determined by UV spectroscopy, fluorescence spectroscopy, real-time PCR or digital PCR (Nakayama et al., 2016; Robin et al., 2016). Although some of these methods offer high accuracy (Robin et al., 2016), identical library size across samples cannot be achieved. Therefore, normalization of read counts will remain inevitable for comparative analyses requiring equal library size. Here we introduce a normalization method for biological count data called scaling with ranked subsampling (SRS).

## Methods

### Normalization methods

#### Rarefying

Rarefying was conducted using the ‘rrarefy’-function in the ‘vegan’ R-package v2.5-6 (Oksanen et al., 2019). The function randomly subsamples OTU counts within each library without replacement until the selected number of counts C_min_ is achieved. Rarefying was performed in the R environment v3.6.1 (R Core Team, 2017).

**Figure 1:**
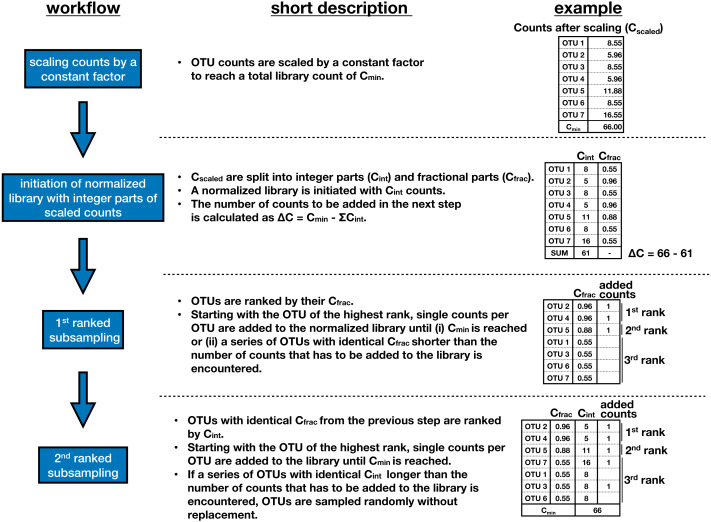
Workflow of scaling with ranked subsampling (SRS). SRS consists of two steps. In the first step, the counts for all OTUs (operational taxonomic untis) are divided by a scaling factor chosen in such a way that the sum of the scaled counts (C_scaled_ with integer or non-integer values) equals C_min_. In the second step, the non-integer count values are converted into integers by an algorithm that we dub ranked subsampling. The scaled count C_scaled_ for each OTU is split into the integer part C_int_ by truncating the digits after the decimal separator (C_int_ = floor(C_scaled_)) and the fractional part C_frac_ (C_frac_ = C_scaled_ − C_int_). Since ΣC_int_ ≤ C_min_, additional ΔC = C_min_ − ΣC_int_ counts have to be added to the library to reach the total count of C_min_. This is achieved as follows. OTUs are ranked in the descending order of their C_frac_ values. Beginning with the OTU of the highest rank, single count per OTU is added to the normalized library until the total number of added counts reaches ΔC and the sum of all counts in the normalized library equals C_min_. When the lowest C_frag_ involved in picking ΔC counts is shared by several OTUs, the OTUs used for adding a single count to the library are selected in the order of their C_int_ values. This selection minimizes the effect of normalization on the relative frequencies of OTUs. OTUs with identical C_frag_ as well as C_int_ are sampled randomly without replacement.

#### Scaling with ranked subsampling (SRS)

The normalization by SRS reduces the number of counts in each sample in such a way that (i) the total count equals C_min_, (ii) each removed OTU is less or equally abundant than any preserved OTU, and (iii) the relative frequencies of OTUs remaining in the sample after normalization are as close as possible to the frequencies in the original sample. The algorithm consists of two steps (Fig. 1). In the first step, the counts for all OTUs are divided by a scaling factor chosen in such a way that the sum of the scaled counts (C_scaled_ with integer or non-integer values) equals C_min_. The relative frequencies of all OTUs remain unchanged. In the second step, the non-integer count values are converted into integers by an algorithm that we dub ranked subsampling (Fig. 1). The scaled count C_scaled_ for each OTU is split into the integer-part C_int_ by truncating the digits after the decimal separator (C_int_ = floor(C_scaled_)) and the fractional part C_frac_ (C_frac_ = C_scaled_ - C_int_). Since ΣC_int_ ≤ C_min_, additional ΔC = C_min_ - ΣC_int_ counts have to be added to the library to reach the total count of C_min_. This is achieved as follows. OTUs are ranked in the descending order of their C_frac_ values, which lie in the open interval (0, 1). Beginning with the OTU of the highest rank, single count per OTU is added to the normalized library until the total number of added counts reaches ΔC and the sum of all counts in the normalized library equals C_min_. For example, if ΔC = 5 and the seven top C_frac_ values are 0.96, 0.96, 0.88, 0.55, 0.55, 0.55, and 0.55, the following counts are added: a single count for each OTU with C_frac_ of 0.96; a single count for the OTU with C_frac_ of 0.88; and a single count each for two OTUs among those with C_frac_ of 0.55. When the lowest C_frag_ involved in picking ΔC counts is shared by several OTUs, the OTUs used for adding a single count to the library are selected in the order of their C_int_values. This selection minimizes the effect of normalization on the relative frequencies of OTUs. OTUs with identical C_frag_ as well as C_int_ are sampled randomly without replacement. An R script that enables the reproduction of this study as well as the test library used in the present study are available at the BonaRes Repository: https://doi.org/10.20387/BONARES-T40J-7VAG.

An implementation of SRS in R is also available at the BonaRes Repository: https://doi.org/10.20387/BONARES-2657-1NP3.

### Test library

The library used for the evaluation of our normalization method represents a soil bacterial microbial community sampled at an agricultural field in Germany. The library is part of a dataset consisting of 60 samples that were sequenced in a single multiplex sequencing run. Total soil DNA was extracted and amplified using the primer pair 341F (5′-CCT ACG GGN GGC WGC AG-3′)/785R (5′-GAC TAC HVG GGT ATC TAA KCC-3′) (Herlemann et al., 2011), and sequenced using the Illumina MiSeq Reagent Kit v3 (2 × 300 bp) (Illumina, San Diego, CA, USA). Data processing in the QIIME 2 environment (v2019.10) (Bolyen et al., 2019) included denoising, merging, chimera filtering, and removing singletons using dada2 (Callahan et al., 2016), clustering amplicon sequence variants, and taxonomic assignment against the SILVA SSU database (release 132) (Quast et al., 2013) using VSEARCH (Rognes et al., 2016). The library consisted of a total of 162,888 counts distributed among 936 OTUs. The library further contained 1,110 OTUs with zero counts, which were included because these OTUs were found in other samples from the same dataset, and thus contained 2,046 OTUs in total. The Shannon diversity of the test library was 5.645.

### Comparison of rarefying and SRS

The 162,888 counts of our test library were normalized to 39 different C_min_ values comprising all integer divisors (factors) 11, 12, 22, 24, 33, 44, 66, 88, 132, 264, 617, 1,234, 1,851, 2,468, 3,702, 4,936, 6,787, 7,404, 13,574, 14,808, 20,361, 27,148, 40,722, 54,296, and 81,444 as well as an exponential series 10, 20, 40, 80, 160, 320, 640, 1,280, 2,560, 5,120, 10,240, 20,480, 40,960, and 81,920. Both rarefying and SRS were used, each with 10,000 replications. Two alpha diversity measures were calculated for each replication: Shannon index *H’* and species richness. The Shannon index was calculated using the ‘diversity’-function in the ‘vegan’ R-package v2.5-6 (Oksanen et al., 2019). Species richness was determined employing the ‘specnumber’-function in the ‘vegan’ R-package v2.5-6. Both alpha diversity measures were calculated in the R environment v3.6.1 (R Core Team, 2017).

The two methods were further compared by artificially raising the total counts of the test library while keeping the relative frequencies of OTUs and alpha diversity constant. To this end, each OTU count was multiplied by 100 and the resulting library of 16,288,800 counts was normalized to 2.5 × 10^5^, 5 × 10^5^, 7.5 × 10^5^, 1 × 10^6^, 2.5 × 10^6^, 5 × 10^6^, 7.5 × 10^6^, and 1 × 10^7^ counts using both rarefying and SRS methods with 10,000 replications each. Two alpha diversity measures were calculated and compared to the known alpha diversity of the original library.

The effect of rarefying and SRS on species composition and their implications for beta diversity were evaluated by determining the Bray–Curtis index of dissimilarity for all pairs of normalized library replications. As in the investigation of the effect of normalization on the alpha diversity described above, the test library was normalized to 39 different C_min_ (see above) using both rarefying and SRS, each with 10,000 replications. The Bray–Curtis index of dissimilarity among all normalized library replications at each C_min_ was determined for both normalization methods. The Bray–Curtis index of dissimilarity was calculated using the ‘vegdist’-function in the ‘vegan’ R-package version 2.5-6 (Oksanen et al., 2019).

In order to examine changes in the relative abundances of OTUs, the test library was normalized to 1 × 10^3^, 1 × 10^4^, and 1 × 10^5^ counts using both rarefying and SRS with 10,000 replications each. The OTUs of the non-normalized library were ranked in a descending order of counts, every 50th OTU starting at the top rank was selected and its relative abundance in all replications of the normalized libraries was determined.

## Results

### Normalization of the test library

**Figure 2:**
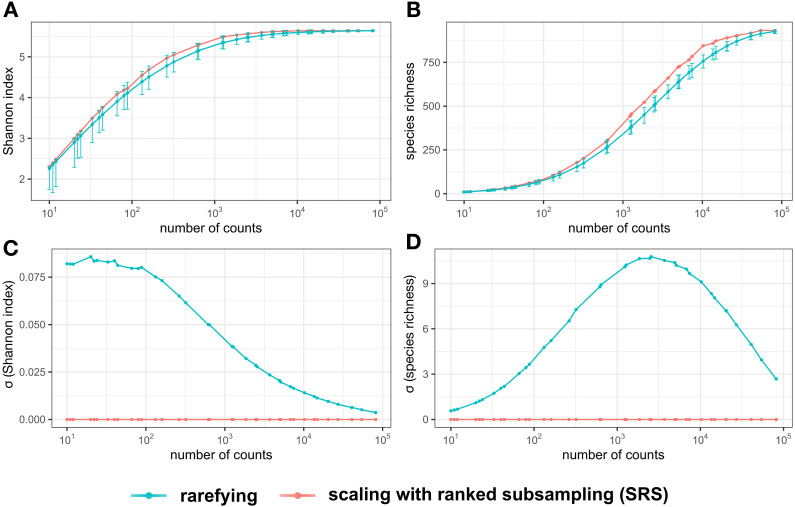
Alpha diversity measures (Shannon index *H’* (A) and species richness (B) of the test library and their standard deviation (*σ*) (C, D) normalized by rarefying or SRS. The sampled numbers of counts were 10, 11, 12, 20, 22, 24, 33, 40, 44, 66, 80, 88, 132, 160, 264, 320, 617, 640, 1,234, 1,280, 1,851, 2,468, 2,560, 3,702, 4,936, 5,120, 6,787, 7,404, 10,240, 13,574, 14,808, 20,361, 20,480, 27,148, 40,722, 40,960, 54,296, 81,444, and 81,920. Means of 10,000 replications (A, B) are represented by points. The minimum and maximum alpha diversity among all 10,000 replications are represented by error bars (A, B). SRS, scaling with ranked subsampling.

SRS showed on average greater alpha diversity as compared to rarefying (Figs. 2A, 2B). The variance of the diversity measures was consistently lower after normalization by SRS as compared to rarefying across all tested C_min_ (Figs. 2C, 2D). No variation of the Shannon index and species richness was observed after SRS. For normalization by rarefying, the variance of the Shannon diversity increased as C_min_ decreased (Figs. 2C, 2D). Variance × the species richness after normalization by rarefying was bell-curve-shaped (Fig. 2D).

### Normalization of a library with artificially raised counts

To confirm our results from the normalization of our test library, we multiplied all counts of our test library by 100 (162,888 × 100 = 16,288,800) -which does not affect alpha diversity- and then normalized the library to different C_min_ above its initial size of 162,888 counts. Ideally, alpha diversity after normalization would remain unchanged in all replications (zero variance). The Shannon diversity of libraries normalized by SRS differed only marginally from the Shannon diversity of the original library (Fig. S1A). Rarefying underestimated or overestimated the Shannon diversity in an extent growing with decreasing C_min_ (Fig. S1A); on the average, Shannon diversity was slightly underestimated. SRS returned the species richness of the original library at all selected C_min_, whereas rarefying underestimated species richness by up to 9 species (Fig. S1B). Libraries normalized by SRS showed no variance for both diversity measures (Figs. S1C, S1D). After rarefying, the variance increased with decreasing C_min_ for both diversity measures (Figs. S1C, S1D).

### Effect of normalization on species composition and implications for beta diversity

**Figure 3:**
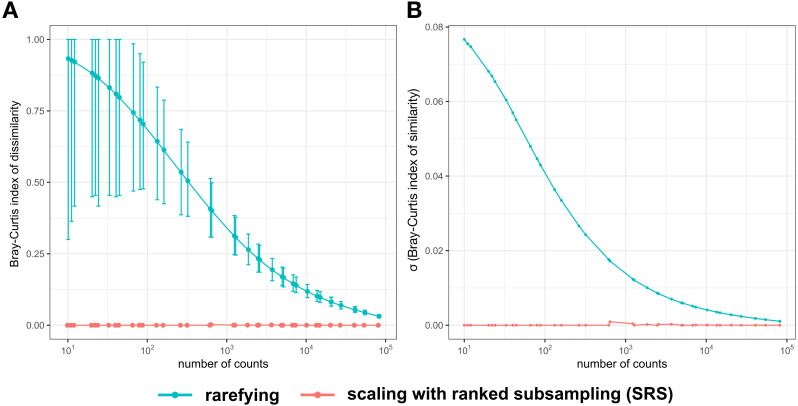
Bray–Curtis index of dissimilarity (A) among 10,000 replications of the normalized test library and its standard deviation (*σ*) (B) normalized by rarefying or SRS. The sampled numbers of counts were 10, 11, 12, 20, 22, 24, 33, 40, 44, 66, 80, 88, 132, 160, 264, 320, 617, 640, 1,234, 1,280, 1,851, 2,468, 2,560, 3,702, 4,936, 5,120, 6,787, 7,404, 10,240, 13,574, 14,808, 20,361, 20,480, 27,148, 40,722, 40,960, 54,296, 81,444, and 81,920. Means of 10,000 replications (A) are represented by points. The minimum and maximum dissimilarity among all 10,000 replications are represented by error bars (A). SRS, scaling with ranked subsampling.

The species composition was evaluated by determining the beta diversity as the Bray–Curtis index of dissimilarity among replications of normalized libraries. Ideally, the index of dissimilarity among replications of the same library would be zero, corresponding to identical species composition. Across 10,000 replications of normalized libraries by rarefying, Bray–Curtis dissimilarity values above 0.5 were observed for the lowest 17 C_min_ (10, 11, 12, 20, 22, 24, 33, 40, 44, 66, 80, 88, 132, 160, 264, 320, and 617 counts) (Fig. 3A). From these 17 C_min_, the index of dissimilarity for some pairs of libraries normalized by rarefying to the first nine C_min_ values was one, which showed that some replications did not share any OTUs. After rarefying, the variance in species composition increased with decreasing C_min_, whereas this was not observed for SRS (Fig. 3B). Differences in species composition among replications of libraries normalized by SRS were only observed when random subsampling of OTUs with the lowest rank of C_frac_ was necessary. For SRS, the maximum dissimilarity found across all replications of all C_min_ was 3.125 × 10^−3^ (found at C_min_ of 640) (Fig. 3A).

### Evaluation of the relative abundance of OTUs

**Figure 4:**
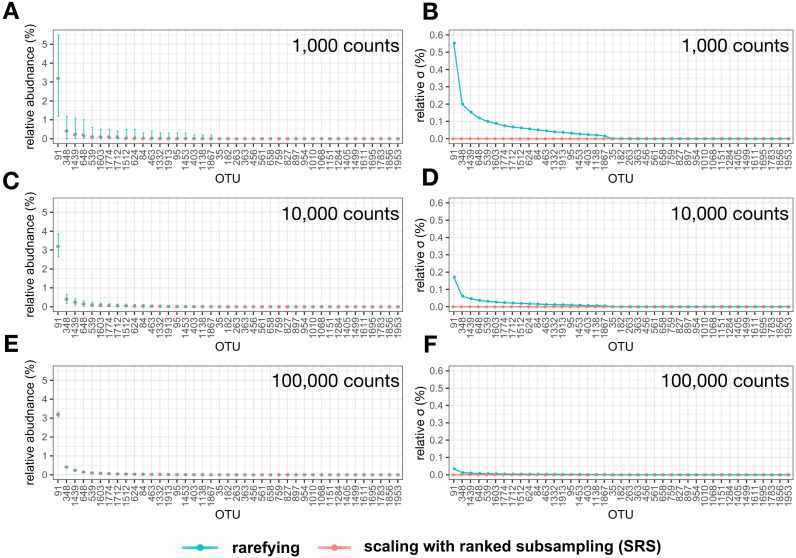
Relative abundance (%) of selected operational taxonomic units (OTUs) at varying library size (A, C, E) and their standard deviation (*σ*) (B, D, F) normalized by rarefying or SRS. The sampled number of counts were 1 × 10^3^, 1 × 10^4^, and 1 × 10^5^. Means of 10,000 replications (A, C, E) are represented by points. The minimum and maximum relative abundance among all OTUs in all 10,000 replications are represented by error bars (A, C, E). SRS, scaling with ranked subsampling.

All OTUs above zero counts in the test library showed varying relative abundance among replications of libraries normalized by rarefying to all three selected C_min_ (1,000, 10,000, and 100,000) (Fig. 4). In contrast, the maximum standard deviation after normalization by SRS amounted to 0.001% relative abundance (OTU ‘1913’ at a C_min_ of 1 × 10^5^ counts) (Figs. 4E–4F). When normalized by rarefying to 1,000 counts, the relative abundance of our most abundant OTU (3.186% relative abundance in the non-normalized library) varied by factor of 4.6 (1.2 to 5.5% relative abundance) (Fig. 4A). Furthermore, our 51st most abundant OTU (OTU ‘348’; 0.404% relative abundance in the non-normalized library) was removed from some normalized libraries, whereas it reached 1.2% relative abundance in other replications after rarefying to 1,000 counts (Fig. 4A). Overall, the variance in relative abundance increased with decreasing C_min_ when rarefying was used, whereas this was not observed for SRS (Fig. 4).

## Discussion

It is well established that primer choice and library preparation can cause biases in microbiome studies that use NGS technologies (Van Dijk, Jaszczyszyn & Thermes, 2014; Schirmer et al., 2015; Tedersoo & Lindahl, 2016). In addition, a number of studies reported that the choice of bioinformatic tools used to process the data can affect the results (*cf.* Plummer et al., 2015; Allali et al., 2017; López-García et al., 2018; Siegwald et al., 2019). Among these tools, the normalization of microbiome count data is much debated. Rarefying has become the standard procedure for normalization (Weiss et al., 2017), although it is statistically inadmissible (McMurdie & Holmes, 2014). In the present study, we introduced SRS as an alternative to rarefying.

Our results demonstrated that SRS has greater reproducibility and accuracy than rarefying when alpha diversity measures (Shannon index *H’* and species richness) were investigated (Fig.2, S1). This was particularly true when the library size differed by multiple orders of magnitude (Fig. 2, S1), which is not uncommon in microbiome studies (McMurdie & Holmes, 2014). Additionally, we observed a strong variation in the relative abundance of OTUs among library replicates normalized by rarefying (Fig. 4). Again, the variance increased with the reduction of the library size (Fig. 4). Rarefying uses random subsampling without replacement which follows the hypergeometric distribution (Simberloff, 1972). Therefore, for an OTU occurring with the frequency f in the original library with N counts, the variance (var) of its abundance a after rarefying to C_min_ is:

\begin{eqnarray*}\mathrm{var} \left( \mathrm{a} \right) ={\mathrm{C}}_{\mathrm{min}}\times \mathrm{f} \times (1-\mathrm{f})\times \frac{(\mathrm{N}-{\mathrm{C}}_{\mathrm{min}})}{(\mathrm{N}-1)} \end{eqnarray*}

For the most abundant OTU in Fig. 4 and C_min_ of 1,000, var(a) equals 30.65, corresponding to a relative standard deviation of 0.554% (*cf.* Fig. 4B). This variance results from a subsampling error incurred by rarefying. SRS largely eliminates the subsampling error.

The purpose of ranking OTUs in the last step of SRS in the order of their C_frag_ and if necessary by C_int_ is to minimize distortion of the species/OTU composition. When OTUs with identical C_frag_ values were picked randomly instead of according to the order of their C_int_, the variance of alpha diversity in libraries normalized to the same C_min_ slightly increased but never reached values comparable to rarefying (Fig. S2).

Due to the law of large numbers, the variance of relative frequencies of OTUs, alpha diversity measures and other parameters after rarefying are expected to grow with decreasing C_min_. Figure 2D shows, however, that the variance of species richness reaches a maximum at a medium library size, asymptotically approaching zero at both very large and very small libraries (in Fig. S1D this effect is not apparent because only libraries of relatively large size have been analyzed). The drop of variance of species richness for small libraries is caused by a systematic error due to normalization to low counts. With the diminishing library size, the number of different OTUs that can be obtained by random subsampling declines; the variance in species richness declines accordingly. Concomitantly, differences in species composition among replicates of libraries normalized by rarefying are expected to grow with decreasing library size. Comparison of libraries normalized by rarefying to the same size confirmed this expectation (Fig. 3). In contrast, the variation in species composition among libraries normalized by SRS was either zero or negligibly low (Fig. 3). The reproducibility of data normalization and the preservation of the original community structure (OTU frequencies) is crucial for the determination of beta diversity among samples.

Our results support the conclusion of McMurdie & Holmes (2014) that rarefying should not be used to normalize microbiome count data. The reason is that random subsampling is the source of variance, which is superimposed on the biological and technical variance. The use of random subsampling in SRS is limited to a fraction of counts that have to be added to the sum of counts scaled and rounded down to integers in order to reach the desired library size. A complex combination of circumstances has to occur for random subsampling to be used in SRS: several OTUs have to share both the integer part and the decimal fraction of their scaled frequencies; in a list of OTUs ranked by frequencies, these OTUs have to appear before the desired total number of counts is reached; and the number of counts that is needed to fill the normalized library is lower than the number of these OTUs. As long as at least one of these conditions is not fulfilled, SRS does not use random subsampling with replacement and replicates of the normalized library are identical. Zero variance of diversity measures for replicates of most libraries in Figs. 2 to 4 is the consequence. If random subsampling is used, the relative abundance of the affected OTUs will be vary by at most a single count. As a consequence, the relative abundance of a rare OTU will be affected more than the relative abundance of a dominant OTU. Therefore, the effect of random subsampling in SRS is expected to be negligible in studies with a library size above 1,000 counts, unless some OTUs are removed while other are kept. Principally, libraries with a high proportion of rare OTUs cannot be normalized to lower integer counts in such a way that all OTUs as well as their frequencies are preserved. In analogy to quantization error in signal processing, no mathematical procedure can circumvent the loss of information due to downscaling counts to integer values. On this background, we believe that SRS is currently the most adequate method for the normalization of species count data and OTU libraries representing microbial communities.

## Conclusion

SRS method for the normalization of species count data minimizes the subsampling error. In contrast to rarefying, common parameters assessed in studies of biodiversity and population structure (alpha diversity, species composition, and relative abundance of OTUs) calculated from data normalized by SRS were highly reproducible and the original community structure (OTU frequencies) was preserved. We therefore propose SRS for the normalization of biological count data.

##  Supplemental Information

10.7717/peerj.9593/supp-1 Supplemental Information 1 Alpha diversity measures (Shannon index *H*′ (A) and species richness (B)) of the test library with artificially raised counts and their standard deviation (*σ* ) (C, D) normalized by rarefying or SRS The sampled number of counts were 2.5 × 10^5^, 5 × 10^5^, 7.5 × 10^5^, 1 × 10^6^, 2.5 × 10^6^, 5 × 10^6^, 7.5 × 10^6^, and 1 × 10^7^. Means of 10,000 replications (A, B) are represented by points. The minimum and maximum alpha diversity among all 10,000 replications are represented by error bars (A, B). SRS, scaling with ranked subsampling.

10.7717/peerj.9593/supp-2 Supplemental Information 2 Alpha diversity measures (Shannon index *H*′ (A) and species richness (B)) and their standard deviation (*σ*) (C, D) normalized by rarefying or SRS with random picking of OTUs with identical C_frag_ OTUs with identical C_frag_ values were picked randomly without replacement in SRS. The sampled numbers of counts were 10, 11, 12, 20, 22, 24, 33, 40, 44, 66, 80, 88, 132, 160, 264, 320, 617, 640, 1,234, 1,280, 1,851, 2,468, 2,560, 3,702, 4,936, 5,120, 6,787, 7,404, 10,240, 13,574, 14,808, 20,361, 20,480, 27,148, 40,722, 40,960, 54,296, 81,444, and 81,920. Means of 10,000 replications (A, B) are represented by points. The minimum and maximum alpha diversity among all 10,000 replications are represented by error bars (A, B). SRS, scaling with ranked subsampling, OTU, operational taxonomic unit.

## Additional Information and Declarations

### Data Availability

The following information was supplied regarding data availability: An R script that enables the reproduction of this study as well as the test library used in the present study are available at the BonaRes Repository: https://doi.org/10.20387/BONARES-T40J-7VAG. An implementation of SRS in R is also available at the BonaRes Repository: https://doi.org/10.20387/BONARES-2657-1NP3.
